# Correction to: Characteristics and prognosis of bloodstream infection in patients with COVID‑19 admitted in the ICU: an ancillary study of the COVID‑ICU study

**DOI:** 10.1186/s13613-022-00979-w

**Published:** 2022-01-11

**Authors:** Nicolas Massart, Virginie Maxime, Pierre Fillatre, Keyvan Razazi, Alexis Ferré, Pierre Moine, Francois Legay, Guillaume Voiriot, Marlene Amara, Francesca Santi, Saad Nseir, Stephanie Marque-Juillet, Rania Bounab, Nicolas Barbarot, Fabrice Bruneel, Charles-Edouard Luyt, the COVID ICU Bacteremia Study Group on behalf of the COVID-ICU Investigators, Tài Pham, Arthur Pavot, Xavier Monnet, Christian Richard, Alexandre Demoule, Martin Dres, Julien Mayaux, Alexandra Beurton, Cédric Daubin, Richard Descamps, Aurélie Joret, Damien Du Cheyron, Frédéric Pene, Jean-Daniel Chiche, Mathieu Jozwiak, Paul Jaubert, Guillaume Voiriot, Muriel Fartoukh, Marion Teulier, Clarisse Blayau, Laetitia Bodenes, Nicolas Ferriere, Johann Auchabie, Anthony Le Meur, Sylvain Pignal, Thierry Mazzoni, Jean-Pierre Quenot, Pascal Andreu, Jean-Baptiste Roudau, Marie Labruyère, Saad Nseir, Sébastien Preau, Julien Poissy, Daniel Mathieu, Sarah Benhamida, Rémi Paulet, Nicolas Roucaud, Martial Thyrault, Florence Daviet, Sami Hraiech, Gabriel Parzy, Aude Sylvestre, Sébastien Jochmans, Anne-Laure Bouilland, Mehran Monchi, Marc Danguy des Déserts, Quentin Mathais, Gwendoline Rager, Pierre Pasquier, Jean Reignier, Amélie Seguin, Charlotte Garret, Emmanuel Canet, Jean Dellamonica, Clément Saccheri, Romain Lombardi, Yanis Kouchit, Sophie Jacquier, Armelle Mathonnet, Mai-Ahn Nay, Isabelle Runge, Frédéric Martino, Laure Flurin, Amélie Rolle, Michel Carles, Rémi Coudroy, Arnaud W. Thille, Jean-Pierre Frat, Maeva Rodriguez, Pascal Beuret, Audrey Tientcheu, Arthur Vincent, Florian Michelin, Fabienne Tamion, Dorothée Carpentier, Déborah Boyer, Christophe Girault, Valérie Gissot, Stéphan Ehrmann, Charlotte Salmon Gandonniere, Djlali Elaroussi, Agathe Delbove, Yannick Fedun, Julien Huntzinger, Eddy Lebas, Grâce Kisoka, Céline Grégoire, Stella Marchetta, Bernard Lambermont, Laurent Argaud, Thomas Baudry, Pierre-Jean Bertrand, Auguste Dargent, Christophe Guitton, Nicolas Chudeau, Mickaël Landais, Cédric Darreau, Alexis Ferre, Antoine Gros, Guillaume Lacave, Fabrice Bruneel, Mathilde Neuville, JérômeDevaquet, Guillaume Tachon, Richard Gallo, Riad Chelha, Arnaud Galbois, Anne Jallot, Ludivine Chalumeau Lemoine, Khaldoun Kuteifan, Valentin Pointurier, Louise-Marie Jandeaux, Joy Mootien, Charles Damoisel, Benjamin Sztrymf, Matthieu Schmidt, Alain Combes, Juliette Chommeloux, Charles Edouard Luyt, Frédérique Schortgen, Leon Rusel, Camille Jung, Florent Gobert, Damien Vimpere, Lionel Lamhaut, Bertrand Sauneuf, Liliane Charrrier, Julien Calus, Isabelle Desmeules, Benoît Painvin, Jean-Marc Tadie, Vincent Castelain, Baptiste Michard, Jean-Etienne Herbrecht, Mathieu Baldacini, Nicolas Weiss, Sophie Demeret, Clémence Marois, Benjamin Rohaut, Pierre-Henri Moury, Anne-Charlotte Savida, Emmanuel Couadau, Mathieu Série, Nica Alexandru, Cédric Bruel, Candice Fontaine, Sonia Garrigou, Juliette Courtiade Mahler, Maxime Leclerc, Michel Ramakers, Pierre Garçon, Nicole Massou, Ly Van Vong, Juliane Sen, Nolwenn Lucas, Franck Chemouni, Annabelle Stoclin, Alexandre Avenel, Henri Faure, Angélie Gentilhomme, Sylvie Ricome, Paul Abraham, Céline Monard, Julien Textoris, Thomas Rimmele, Florent Montini, Gabriel Lejour, Thierry Lazard, Isabelle Etienney, Younes Kerroumi, Claire Dupuis, Marine Bereiziat, Elisabeth Coupez, François Thouy, Clément Hoffmann, Nicolas Donat, Anne Chrisment, Rose-Marie Blot, Antoine Kimmoun, Audrey Jacquot, Matthieu Mattei, Bruno Levy, Ramin Ravan, Loïc Dopeux, Jean-Mathias Liteaudon, Delphine Roux, Brice Rey, Radu Anghel, Deborah Schenesse, Vincent Gevrey, Jermy Castanera, Philippe Petua, Benjamin Madeux, Otto Hartman, Michael Piagnerelli, Anne Joosten, Cinderella Noel, Patrick Biston, Thibaut Noel, Gurvan L. E. Bouar, Messabi Boukhanza, Elsa Demarest, Marie-France Bajolet, Nathanaël Charrier, Audrey Quenet, Cécile Zylberfajn, Nicolas Dufour, Buno Mégarbane, Sébastian Voicu, Nicolas Deye, Isabelle Malissin, François Legay, Matthieu Debarre, Nicolas Barbarot, Pierre Fillatre, Bertrand Delord, Thomas Laterrade, Tahar Saghi, Wilfried Pujol, Pierre Julien Cungi, Pierre Esnault, Mickael Cardinale, Vivien Hong Tuan Ha, Grégory Fleury, Marie-Ange Brou, Daniel Zafimahazo, David Tran-Van, Patrick Avargues, Lisa Carenco, Nicolas Robin, Alexandre Ouali, Lucie Houdou, Christophe Le Terrier, Noémie Suh, Steve Primmaz, Jérome Pugin, Emmanuel Weiss, Tobias Gauss, Jean-Denis Moyer, Catherine Paugam Burtz, Béatrice La Combe, Rolland Smonig, Jade Violleau, Pauline Cailliez, Jonathan Chelly, Antoine Marchalot, Cécile Saladin, Christelle Bigot, Pierre-Marie Fayolle, Jules Fatséas, Amr Ibrahim, Dabor Resiere, Rabih Hage, Clémentine Cholet, Marie Cantier, Pierre Trouiler, Philippe Montravers, Brice Lortat-Jacob, Sebastien Tanaka, Alexy Tran Dinh, Jacques Duranteau, Anatole Harrois, Guillaume Dubreuil, Marie Werner, Anne Godier, Sophie Hamada, Diane Zlotnik, Hélène Nougue, Armand Mekontso-Dessap, Guillaume Carteaux, Keyvan Razazi, Nicolas De Prost, Nicolas Mongardon, Meriam Lamraoui, Claire Alessandri, Quentin de Roux, Charles de Roquetaillade, Benjamin G. Chousterman, Alexandre Mebazaa, Etienne Gayat, Marc Garnier, Emmanuel Pardo, LeaSatre-Buisson, Christophe Gutton, Elise Yvin, Clémence Marcault, Elie Azoulay, Michael Darmon, Hafid Ait Oufella, Geoffroy Hariri, Tomas Urbina, Sandie Mazerand, Nicholas Heming, Francesca Santi, Pierre Moine, Djillali Annane, Adrien Bouglé, Edris Omar, Aymeric Lancelot, Emmanuelle Begot, Gaétan Plantefeve, Damien Contou, Hervé Mentec, Olivier Pajot, Stanislas Faguer, Olivier Cointault, Laurence Lavayssiere, Marie-Béatrice Nogier, Matthieu Jamme, Claire Pichereau, Jan Hayon, Hervé Outin, François Dépret, Maxime Coutrot, Maité Chaussard, Lucie Guillemet, Pierre Goffin, Romain Thouny, Julien Guntz, Laurent Jadot, Romain Persichini, Vanessa Jean-Michel, Hugues Georges, Thomas Caulier, Gaël Pradel, Marie-Hélène Hausermann, Thi My Hue Nguyen-Valat, Michel Boudinaud, Emmanuel Vivier, SylvèneRosseli, Gaël Bourdin, Christian Pommier, Marc Vinclair, Simon Poignant, Sandrine Mons, Wulfran Bougouin, Franklin Bruna, Quentin Maestraggi, Christian Roth, Laurent Bitker, François Dhelft, Justine Bonnet-Chateau, Mathilde Filippelli, Tristan Morichau-Beauchant, Stéphane Thierry, Charlotte Le Roy, Mélanie Saint Jouan, Bruno Goncalves, Aurélien Mazeraud, Matthieu Daniel, Tarek Sharshar, Cyril Cadoz, RostaneGaci, Sébastien Gette, Guillaune Louis, Sophe-Caroline Sacleux, Marie-Amélie Ordan, Aurélie Cravoisy, Marie Conrad, Guilhem Courte, Sébastien Gibot, Younès Benzidi, Claudia Casella, Laurent Serpin, Jean-Lou Setti, Marie-Catherine Besse, Anna Bourreau, Jérôme Pillot, Caroline Rivera, Camille Vinclair, Marie-Aline Robaux, Chloé Achino, Marie-Charlotte Delignette, Tessa Mazard, Frédéric Aubrun, Bruno Bouchet, Aurélien Frérou, Laura Muller, Charlotte Quentin, Samuel Degoul, Xavier Stihle, Claude Sumian, Nicoletta Bergero, Bernard Lanaspre, Hervé Quintard, Eve Marie Maiziere, Pierre-Yves Egreteau, Guillaume Leloup, Florin Berteau, Marjolaine Cottrel, Marie Bouteloup, Matthieu Jeannot, Quentin Blanc, Julien Saison, Isabelle Geneau, Romaric Grenot, Abdel Ouchike, Pascal Hazera, Anne-Lyse Masse, Suela Demiri, Corinne Vezinet, Elodie Baron, Deborah Benchetrit, Antoine Monsel, Grégoire Trebbia, Emmanuelle Schaack, Raphaël Lepecq, Mathieu Bobet, Christophe Vinsonneau, Thibault Dekeyser, Quentin Delforge, Imen Rahmani, Bérengère Vivet, Jonathan Paillot, Lucie Hierle, Claire Chaignat, Sarah Valette, Benoït Her, Jennifier Brunet, Mathieu Page, Fabienne Boiste, Anthony Collin, Florent Bavozet, Aude Garin, Mohamed Dlala, KaisMhamdi, Bassem Beilouny, Alexandra Lavalard, Severine Perez, Benoit Veber, Pierre-Gildas Guitard, Philippe Gouin, Anna Lamacz, Fabienne Plouvier, Bertrand P. Delaborde, Aïssa Kherchache, Amina Chaalal, Jean-Damien Ricard, Marc Amouretti, Santiago Freita-Ramos, Damien Roux, Jean-Michel Constantin, Mona Assefi, Marine Lecore, Agathe Selves, Florian Prevost, Christian Lamer, Ruiying Shi, Lyes Knani, Sébastien Pili Floury, Lucie Vettoretti, Michael Levy, Lucile Marsac, Stéphane Dauger, Sophie Guilmin-Crépon, Hadrien Winiszewski, Gael Piton, Thibaud Soumagne, Gilles Capellier, Jean-Baptiste Putegnat, Frédérique Bayle, Maya Perrou, Ghyslaine Thao, Guillaume Géri, Cyril Charron, Xavier Repessé, Antoine Vieillard-Baron, Mathieu Guilbart, Pierre-Alexandre Roger, Sébastien Hinard, Pierre-Yves Macq, Kevin Chaulier, Sylvie Goutte, Patrick Chillet, Anaïs Pitta, Barbara Darjent, Amandine Bruneau, Sigismond Lasocki, Maxime Leger, Soizic Gergaud, Pierre Lemarie, Nicolas Terzi, Carole Schwebel, Anaïs Dartevel, Louis-Marie Galerneau, Jean-Luc Diehl, Caroline Hauw-Berlemont, Nicolas Péron, Emmanuel Guérot, Abolfazl Mohebbi Amoli, Michel Benhamou, Jean-Pierre Deyme, Olivier Andremont, Diane Lena, Julien Cady, Arnaud Causeret, Arnaud De La Chapelle, Christophe Cracco, Stéphane Rouleau, David Schnell, Camille Foucault, Cécile Lory, Thibault Chapelle, Vincent Bruckert, Julie Garcia, Abdlazize Sahraoui, Nathalie Abbosh, Caroline Bornstain, Pierre Pernet, Florent Poirson, Ahmed Pasem, Philippe Karoubi, Virginie Poupinel, Caroline Gauthier, François Bouniol, Philippe Feuchere, Anne Heron, Serge Carreira, Malo Emery, Anne Sophie Le Floch, Luana Giovannangeli, Nicolas Herzog, Christophe Giacardi, Thibaut Baudic, Chloé Thill, Said Lebbah, Jessica Palmyre, Florence Tubach, David Hajage, Nicolas Bonnet, Nathan Ebstein, Stéphane Gaudry, Yves Cohen, Julie Noublanche, Olivier Lesieur, Arnaud Sément, Isabel Roca-Cerezo, Michel Pascal, Nesrine Sma, Gwenhaël Colin, Jean-Claude Lacherade, Gauthier Bionz, Natacha Maquigneau, Pierre Bouzat, Michel Durand, Marie-Christine Hérault, Jean-Francois Payen

**Affiliations:** 1 Service de Réanimation, CH de St BRIEUC, 10, rue Marcel Proust, 22000 Saint-Brieuc, France; 2 Surgical and Medical Intensive Care Unit Hôpital, Raymond Poincaré, 9230 Garches, France; 3 AP-HP, Hôpitaux Universitaires Henri-Mondor, Service de Médecine Intensive Réanimation, 94010 Créteil, France; 4 Univ Paris Est Créteil, INSERM, IMRB, 94010 Créteil, France; 5 Université Paris Est Créteil, Faculté de Médecine de Créteil, IMRB, GRC CARMAS, 94010 Créteil, France; 6 Service de Réanimation/USC, Hôpital Mignot, Centre hospitalier de Versailles, 177 rue de Versailles, 78150 Le Chesnay, France; 7 Service de Médecine Intensive Réanimation, Hôpital Tenon, Assistance Publique-Hôpitaux de Paris, Sorbonne Université, and Groupe de Recherche Clinique CARMAS, Collegium Galilée, Créteil, France; 8 Centre de Réanimation, CHU de Lille, 59000 Lille, France; 9 INSERM U1285, Université de Lille, CNRS, UMR 8576 – UGSF – Unité de Glycobiologie Structurale et Fonctionnelle, 59000 Lille, France; 10 Service de Biologie (Unité de Microbiologie), Hôpital Mignot, Centre Hospitalier de Versailles, 177 rue de Versailles, 78150 Le Chesnay, France; 11 Service de Médecine Intensive Réanimation, Institut de Cardiologie, Assistance Publique–Hôpitaux de Paris (APHP), Sorbonne-Université, Hôpital Pitié–Salpêtrière, and Sorbonne Université, INSERM, UMRS_1166-ICAN Institute of Cardiometabolism and Nutrition, 47–83, Boulevard de l’Hôpital, 75651 Paris, France

## Correction to: Annals of Intensive Care (2021) 11:183 10.1186/s13613-021-00971-w

Unfortunately, in the original publication of the article [1], heading of third column in Table 4, was mistakenly mentioned as “*n* = 537” instead of “Patients without BSI* n* = 537”. The corrected Table 4 is given in this erratum.

**Table 4:** Outcomes among 537 patients with bloodstream infection and their 537 propensity-matched patients without bloodstream infection

	Patients with BSI *n* = 537	Patients without BSI *n *= 537	*p*-value
Length of stay in ICU, days	24 [15–36]	13 [6–25]	< 0.001
Length of stay in hospital, days	40 [27–58]	24 [13–40]	< 0.001
ICU-free days at day 90, days	52 [0–74]	65 [0–81]	< 0.001
Ventilator-free days at day 90, days	61 [0–81]	70 [0–88]	< 0.001
Death at day 90	212 (39)	178 (33)	0.036

Data are expressed as median [interquartile range] or *n* (%)

*BSI* bloodstream infection, *ICU* intensive care unit

The original article has been corrected.
